# Neonatal necrotizing enterocolitis complicated by glutaric acidemia type II: a case report

**DOI:** 10.3389/fped.2025.1392927

**Published:** 2025-02-03

**Authors:** Yuli Zhang, Longfei Chen, Miao Duan

**Affiliations:** ^1^ Department of Neonate, The Third Affiliated Hospital of Zunyi Medical University, The First People’s Hospital of Zunyi, Zunyi, China; ^2^ Department of Neonatology, People’s Hospital of Xiuwen County, Guiyang, China; ^3^ Department of Hepatobiliary, The Third Affiliated Hospital of Zunyi Medical University, The First People’s Hospital of Zunyi, Zunyi, China

**Keywords:** glutaric, acidemia neonatal, necrotizing enterocolitis, preterm, infant

## Abstract

Glutaric acidemia type II (GAII) is an autosomal recessive genetic metabolic disorder associated with mitochondrial dysfunction, characterized by multiple acyl-CoA dehydrogenase deficiency that affects fatty acid metabolism. Necrotizing enterocolitis (NEC) represents a severe inflammatory condition affecting premature neonates. This report describes a case involving a male preterm infant born at a gestation of 30^ + 1^ weeks who developed NEC complicated by GAII. On the eighth day of life, the patient exhibited abdominal distension and vomiting. Diagnostic imaging, including abdominal B-ultrasound and x-ray, revealed thickened bowel walls, multiple intestinal pneumatosis, and partial intestinal dilation, consistent with NEC. Subsequent recurrent episodes of acidosis, hyperlactacidemia, and hypoglycemia were observed. Diagnosis of GAII was confirmed through tandem mass spectrometry analysis of a blood sample. Genetic metabolic diseases may complicate or mimic common infections, leading to potential misdiagnosis. A differential diagnosis of GAII should be considered when active anti-infective treatments fail.

## Introduction

Glutaric acidemia type II (GAII), also known as multiple acyl-CoA dehydrogenase deficiency, results from defects in the electron transfer flavoprotein (ETF) or ETF dehydrogenase (ETFDH). These proteins are crucial for electron transfer (1), and their deficiencies impair the metabolism of fatty and amino acids (1). GAII is categorized based on the age of onset and clinical manifestations into three types: neonatal onset with congenital anomalies (type I), neonatal onset without anomalies (type II), and late onset (type III) (2). The neonatal onset form is particularly severe, often associated with high mortality and presenting symptoms such as hepatomegaly, non-ketotic hypoglycemia, metabolic acidosis, hypotonia, and cardiomyopathy (2, 3).

Necrotizing enterocolitis (NEC) is a severe inflammatory condition in premature neonates with a complex, poorly understood pathogenesis. According to Bell's classification, it can be categorized into suspected (stage I), moderate (stage II), or severe (stage III) NEC (4). The condition arises from a combination of factors, including prematurity, infections, hypoxic/ischemic insults, and immune responses. Common symptoms include abdominal distension, vomiting, bloody stool, severe shock, and multiple organ failure (5).

To date, the co-occurrence of NEC and GAII has not been documented. This report describes a case of a patient presenting with both NEC and GAII, highlighting the complexity and rarity of this combination.

### Case report

We reported a case of a 30^ + 1^ weeks male preterm infant delivered by cesarean section with clear amniotic fluid and no nuchal cord. The Apgar score was 10 at 1, 5, and 10 min. The birth weight was 1,100 g, within the normal range for a 30^ + 1^ week preterm infant (approximately 1,000–1,500 g) and considered appropriate for gestational age (AGA). Owing to preterm birth with shortness of breath and vomiting (with milk-like vomitus) for half an hour, he was admitted to the neonatal intensive care unit of our hospital on the first day of life. The infant was born to a 33-year-old G5P2 mother who had a history of severe antenatal pre-eclampsia and thrombocytopenia. The mother received limited prenatal care during pregnancy. The mother was given one course of dexamethasone during the pregnancy to promote fetal lung maturity. The mother denied consanguinity and a family history of any genetic diseases.

On physical exam, the infant showed a poor response, nasal alar flap, and the typical appearance of a premature infant. Shortness of breath, irregular rhythm, and positive tri-retraction signs were also noted. The infant's heart rate was 152 beats per minute with a temperature of 36°C. The infant's skin did not show jaundice, ecchymosis, and purpura. Other body parts showed findings consistent with those of a healthy newborn.

Routine blood tests, biochemical examination, and blood gas analysis are shown in Tables 1–3. The infant was given milk for 2 days after birth, including premature infant milk, and nutritional support, such as amino acids and fat emulsion, as well as fluid replacement to maintain internal environment and blood sugar stability. On the eighth day of life, the infant developed abdominal distension, vomiting, and low fever; these concerns were successfully resolved with symptomatic treatment. On the 12th day of life, the patient's heart rate increased rapidly (190–200 bpm). The chest radiograph showed an enlargement of the cardiac shadow. Considering the presence of neonatal sepsis, cefotaxime sodium plus flucloxacillin were started. The feeding amount of breast milk was 10 ml every 3 h. On the 15th day of life, abdominal distension and vomiting still occurred during the fasting period in the patient; meanwhile, increased heart rate and occasional episodes of fever persisted as well. Abdominal B-ultrasound and abdominal x-ray revealed thickened and stiff abdominal and bowel walls, multiple intestinal pneumatosis, and partial intestinal dilation. These findings are consistent with the typical radiographic and ultrasonographic features of NEC, including bowel wall thickening, pneumatosis intestinalis, and portal venous gas (6). The combination of these imaging findings, along with the medical history and clinical signs and symptoms, strongly supported the diagnosis of NEC. Meropenem plus metronidazole was initiated, and other conservative approaches were used, such as dopamine, which can improve circulation. During the treatment, the concentration of hemoglobin and albumin was reduced to 120 g/L and 23.5 g/L, respectively, and the same type of red blood cells and human serum albumin were infused.

**Table 1:** Changes in blood routine parameters of the patient.

Routine blood test	Day age (days)						
	1	15	18	23	25	27	28
WBC (×10^9^/L)	7.42↓	5.47↓	5.22↓	4,68↓	4.34↓	2.57↓	3.49↓
Neutrophil percentage	0.58	0.52	0.46	0.68	0.33	0.37	0.35
RBC_S_ (×10^12^/L)	4.87	3.42↓	3.21↓	3.32↓	3.01↓	3.68↓	3.7↓
Hematocrit (L/L)	0.55	0.34↓	0.32↓	0.32↓	0.27↓	0.32↓	0.32↓
Hemoglobin (g/L)	207	128↓	120↓	117↓	100↓	120↓	122↓
Platelet counts (×10^9^/L)	167	164	91↓	40↓	34↓	21↓	25↓

WBC, total white blood cells; RBCs, total number of red blood cells. ↓, below the reference limit.

**Table 2:** Changes in blood biochemical parameters of the patient.

Blood biochemical examination	Day age (days)					
	1	11	15	21	23	25
Total bilirubin (µmmol/L)	149.6↑			113	117.1	
Indirect bilirubin (µmol/L)	138.4↑	63		62.9	56.7	
Direct bilirubin (µmmol/L)	11.2	27.6		50.1	60.4↑	
Total bile acids (µmmol/L)	14.78	30.3		53.16↑	55.06↑	
Albumin (g/L)	30.8	24.7↓		28.4↓	25.7↓	
Creatine kinase (U/L)	149	41			1,108↑	988↑
Creatine kinase isoenzyme (U/L)	27	18			169↑	89↑
ALT (U/L)	5	3		1		
AST (U/L)	33	13		9		59↑
Sodium ion (mmol/L)	142.28	141.73		135.18	134.22	134.31
Potassium ion (mmol/L)	4.15	4.86		3.15↓	6.27	5.19
calcium ion (mmol/L)	2.17	2.78		1.91	1.64↓	1.3↓
CRP (mg/L)	2.2	1	<10	2.7		11.6↑
Procalcitonin (ng/ml)			0.79		0.47	
IL-6 (pg/ml)					34.1↑	

↑, above the reference limit; ↓, below the reference limit.

**Table 3:** Changes in results of blood gas analysis.

Blood gas analysis	Day age (days)								
	0	15	19	23	24	25	26	27	28
pH	7.28↓	7.493	7.433	7.318	7.28↓	7.335	7.32	7.32	7.31
PO_2_ (mmHg)	112	50.9↓	56↓	243↑	127.1↑	116.8↑	123.8↑	96.7	58.4↓
PCO_2_ (mmHg)	37.3	32.8	22.4↓	25↓	16.2↓	24.2↓	19.5↓	35.1	22.5↓
HCO_3_^−^ (mmol/L)	17.2	24.6	14.7↓	12.5↓	7.5↓	12.6↓	10.0↓	17.9↓	11.1↓
BE (mmol/L)	−8.73↓	1.84	−8.10↓	−12.06↓	−16.9↓	−1.89↑	−14.48↓	−7.33↓	−13.54↓
Lactic acid (mmol/L)	1.8	3.4	2.8		14.6↑	8.3↑	7.9↑	10↑	10.2↑
Blood sugar (mmol/L)	1.7↓	3.9		4.6	2.8	2.1↓	2.6	2.7	2.8

↑, above the reference limit; ↓, below the reference limit.

**Figure 1:**
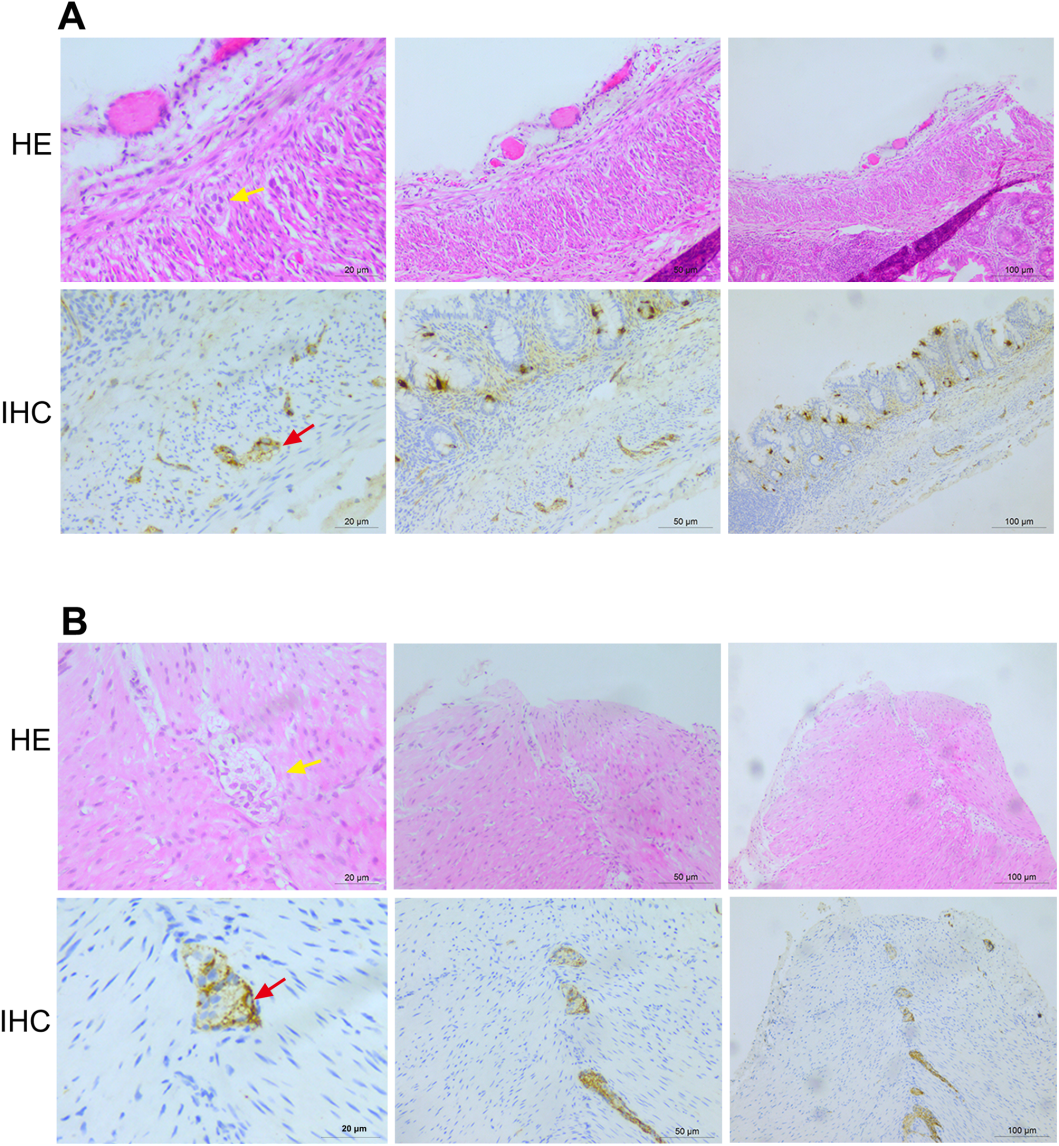
Histopathological examination results after the operation. **(A)** Histopathological examination results of the sigmoid colon show 11 nerve plexuses, with 0–5 ganglia in each plexus. **(B)** Histopathological examination results of the appendix wall show 35 nerve plexuses, with 2–4 ganglia in each plexus. HE, hematoxylin and eosin staining; IHC, immunohistochemistry.

On the 22nd day of life, the conservative approach effect was ineffective. Abdominal distension progressively worsened, and the skin of the entire body appeared gray. Considering the possibility of carnitine deficiency, carnitine was supplemented. Electrolyte disorder (hyponatremia and hypokalemia) occurred at the same time. Abdominal x-ray showed multiple intestinal pneumatosis, partial intestinal dilation, and bowel wall pneumatosis. The patient was diagnosed as having NEC (stage III) and was urgently transferred to the operating room for exploratory laparotomy, multi-segment colonic biopsies, appendectomy, intestinal decompression, and single-end ileostomy. Postoperative pathological examination further confirmed the diagnosis of NEC (Figure 1).

**Figure 2:**
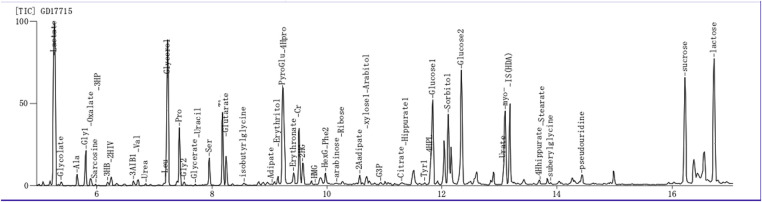
Gas chromatography-mass spectrometry for the detection of urine organic acids. Glutaric acidemia type II with suspected severe metabolic acidosis.

After the operation, the patient presented with recurrent episodes of acidosis, hyperlactic acidemia, and hypoglycemia. Combined with the history and clinical features, genetic metabolic disease was considered. Tandem mass spectrometry analysis of the infant's blood sample showed that the blood ammonia concentration was 88.7 µmol/L (normal range: 18–72 mol/L) (Table 4). On the 28th day of life, metabolic screening showed multiple acyl-CoA dehydrogenase deficiencies GAII, medium- and short-chain fatty acid oxidation abnormalities, and increased blood lactic acid levels (Figure 2). As a result of these findings coupled with the progressive decline of white blood cell and platelet counts, the patient was diagnosed as having GAII, fatty acid oxidation abnormalities, and hyperlactic acidemia. Intravenous nutrition was stopped, and L-carnitine was infused to promote metabolism. Unfortunately, on the 29th day of life, the patient's condition was exacerbated, culminating in death after rescue therapy.

**Table 4:** Tandem mass spectrometry analysis report.

Testing indicators	Normal range	Test result	Remark
Citrulline (Cit)	3.00–25.00	44.43	↑
Cysteine (Cys)	0.10–0.55	0.56	↑
Leucine (Leu)	45.00–300.00	417.05	↑
Proline (Pro)	50.00–400.00	1,091.49	↑
Threonine (Thr)	10.00–90.00	123.89	↑
Citrulline (Cit)/Phenylalanine (Phe) (Cit/Phe)	0.06–0.55	1.53	↑
Leucine (Leu)/Phenylalanine (Phe) (Leu/Phe)	1.50–6.50	14.4	↑
Threonine (Thr)/Phenylalanine (Phe) (Thr/Phe)	0.20–2.50	4.28	↑
C5-Hydroxy (C5-OH)/Free Carnitine (C0) (C5-OH/C0)	0.00–0.02	0.04	↑
C5-Dicarboxylic (C5DC)/C5-Hydroxy (C5-OH) (C5DC/C5-OH)	0.01–2.50	3.32	↑
C5-Dicarboxylic (C5DC)/Carnitine 16 (C16) (C5DC/C16)	0.00–0.10	2.07	↑
Octanoylcarnitine (C8)/Acetylcarnitine (C2) (C8/C2)	0.00–0.02	0.16	↑
Hexadecenoylcarnitine (C16-OH)/Carnitine 16 (C16) (C16-OH/C16)	0.00–0.06	0.03	↑
Tetradecenoylcarnitine (C14:1)/Carnitine 16 (C16) (C14:1/C16)	0.01–0.60	0.9	↑
Hexanoylcarnitine (C6)/Propionylcarnitine (C3) (C6/C3)	0.00–0.12	1.07	↑
Octanoylcarnitine (C8)/Propionylcarnitine (C3) (C8/C3)	0.00–0.15	1.98	↑
Decanoylcarnitine (C10)/Propionylcarnitine (C3) (C10/C3)	0.00–0.20	1.89	↑
Dodecanoylcarnitine (C12)/Propionylcarnitine (C3) (C12/C3)	0.00–0.25	0.49	↑
Butyrylcarnitine (C4)	0.01–0.40	1.42	↑
Isovalerylcarnitine (C5)	0.01–0.25	0.73	↑
Glutarylcarnitine (C5DC)	0.00–0.18	0.75	↑
Hexanoylcarnitine (C6)	0.01–0.08	0.37	↑
Adipylcarnitine (C6DC)	0.00–0.06	0.08	↑
Octanoylcarnitine (C8)	0.00–0.10	0.68	↑
Decanoylcarnitine (C10)	0.01–0.15	0.65	↑
Hexenoylcarnitine (C6:1)	0.01–0.04	0.08	↑
Decenoylcarnitine (C10:1)	0.01–0.15	0.64	↑
Decadienoylcarnitine (C10:2)	0.00–0.03	0.05	↑
Tetradecadienoylcarnitine (C14:2)	0.00–0.08	0.21	↑
Hexadecadienoylcarnitine (C16:2)	0.00–0.03	0.05	↑
(Acetylcarnitine+C2+C3+Hexadecenoylcarnitine (C16)+Stearylcarnitine (C18:1))/Citrulline ((0+2+3+16+18:1)/Cit)	1.00–20.00	0.35	↓
Methionine (Met)/Leucine (Leu) (Met/Leu)	0.05–0.35	0.02	↓
Valine (Val)/Phenylalanine (Phe) (Val/Phe)	1.00–4.00	4.98	↑

↑, above the reference limit; ↓, below the reference limit.

## Discussion

The patient in this case was a low birth weight preterm infant who encountered multiple complications during treatment, including neonatal sepsis, feeding intolerance, and NEC. Initially, NEC was managed conservatively; however, surgical intervention became necessary as the condition escalated to severe NEC (stage III) (7). Post-surgery, the patient's condition did not improve, evidenced by uncontrolled infection, declining white blood cell and platelet counts, and recurrent episodes of metabolic acidosis, hyperlactic acidemia, and hypoglycemia. These symptoms, while typical of GAII, can also overlap with manifestations of NEC (3). Although vomiting, abdominal distention, and feeding intolerance are common symptoms in preterm infants, these nonspecific findings often complicate the differential diagnosis between NEC and GAII (5, 7). However, this case is unique in that the co-existence of NEC and GAII has been reported for the first time. This suggests that the problem is not just distinguishing between NEC and GAII but recognizing that NEC may overlap with GAII in neonates. Therefore, in the face of suspected NEC that does not respond to conventional anti-infective therapy, an underlying metabolic disorder, such as GAII, should be considered and screened accordingly.

This case illustrates the unique co-existence of NEC and GAII, a combination not previously reported, highlighting the critical need to consider the possibility of GAII in patients presenting with NEC. In clinical settings, patients with GAII may present with metabolic acidosis and recurrent hyperprolactinemia. If conventional anti-infective treatments prove ineffective, clinicians should evaluate for GAII. In this instance, the initial symptoms of abdominal distension and vomiting prompted investigations that confirmed NEC via ultrasound and x-ray. Subsequent metabolic disturbances indicated a potential underlying metabolic disorder, leading to the diagnosis of GAII through tandem mass spectrometry and metabolic screening. Early and accurate diagnosis is vital for initiating appropriate interventions that could extend the patient's life (8).

The co-existence of acute conditions, such as NEC with GAII, can exacerbate clinical symptoms due to underlying metabolic disorders. NEC, a severe inflammatory disease in premature infants, involves multiple pathogenic factors, including prematurity, infection, hypoxic/ischemic injury, and immune responses (5, 9). In GAII patients suffering from NEC, metabolic disorders may worsen symptoms such as metabolic acidosis and hyperprolactinemia, contributing to cellular damage via oxidative stress and disruptions in energy metabolism. These metabolic complications are particularly detrimental to the developing brain, showing the importance of comprehensive care in these complex clinical scenarios (10–12).

Although GAII and NEC have almost no co-existence, examining their potential metabolic interactions is crucial. Metabolic disorders in GAII may predispose newborns to increased susceptibility to inflammation and intestinal damage, potentially facilitating the onset of NEC. Conversely, the inflammation and tissue damage associated with NEC can intensify the metabolic disturbances in GAII patients, creating a deleterious cycle that exacerbates both metabolic imbalance and tissue injury. Therefore, in cases of NEC, particularly in newborns presenting with symptoms like metabolic acidosis and hyperprolactinemia, clinicians should maintain a high index of suspicion for GAII and ensure appropriate screening and management are implemented (13).

There are several limitations to this case report, firstly, it is based on the nature of a single case, which limits the generalizability of the conclusions and does not provide statistically significant or broadly applicable clinical guidance. Secondly, due to the severity of the patient's condition, timely cardiac ultrasound examination and postmortem genetic testing were not performed, resulting in a lack of understanding of the underlying etiology and pathophysiological mechanisms, which affected the in-depth discussion of disease associations. Finally, multiple interventions were used in the treatment process, including antibiotic use and surgical treatment, which made it difficult to clearly distinguish which factors directly contributed to the patient's outcome and increased the complexity of the analysis.

## Conclusion

While cases of concurrent GAII and NEC are relatively uncommon, the interplay of their metabolic pathways is significant and warrants attention. In newborns with suspected NEC who do not respond to conventional treatment, particularly those with symptoms such as metabolic acidosis and hyperprolactinemia, clinicians should maintain a high index of suspicion for GAII and ensure appropriate screening and management are implemented. This case, despite the absence of genetic testing and autopsy, provides a critical clinical reference for the potential co-occurrence of these conditions. Future research is essential to further explore the relationship between GAII and NEC, emphasizing the importance of early diagnosis and intervention in GAII to potentially extend patient survival.

## Data Availability

The original contributions presented in the study are included in the article/Supplementary Material, further inquiries can be directed to the corresponding author.
